# Ovarian cancer circulating extracelluar vesicles promote coagulation and have a potential in diagnosis: an iTRAQ based proteomic analysis

**DOI:** 10.1186/s12885-019-6176-1

**Published:** 2019-11-12

**Authors:** Wei Zhang, Peng Peng, Xiaoxuan Ou, Keng Shen, Xiaohua Wu

**Affiliations:** 1 Department of Gynecologic Oncology, Fudan University Shanghai Cancer Center, 270 Dong-an Road, Shanghai, 200032 People’s Republic of China; 2 Department of Obstetrics and Gynecology Peking Union Medical College (PUMC) Hospital, Chinese Academy of Medical Sciences & Peking Union Medical College, Beijing, China

**Keywords:** Epithelial ovarian cancer, Extracellular vesicles, Proteomics, Biomarker, Diagnosis

## Abstract

**Background:**

Circulating extracelluar vesicles (EVs) in epithelial ovarian cancer (EOC) patients emanate from multiple cells. These EVs are emerging as a new type of biomarker as they can be obtained by non-invasive approaches. The aim of this study was to investigate circulating EVs from EOC patients and healthy women to evaluate their biological function and potential as diagnostic biomarkers.

**Methods:**

A quantitative proteomic analysis (iTRAQ) was applied and performed on 10 EOC patients with advanced stage (stage III–IV) and 10 controls. Twenty EOC patients and 20 controls were applied for validation. The candidate proteins were further validated in another 40-paired cohort to investigate their biomarker potential. Coagulation cascades activation was accessed by determining Factor X activity.

**Results:**

Compared with controls, 200 proteins were upregulated and 208 proteins were downregulated in the EOC group. The most significantly involved pathway is complement and coagulation cascades. ApoE multiplexed with EpCAM, plg, serpinC1 and C1q provide optimal diagnostic information for EOC with AUC = 0.913 (95% confidence interval (CI) =0.848–0.957, *p* < 0.0001). Level of activated Factor X was significantly higher in EOC group than control (5.35 ± 0.14 vs. 3.69 ± 0.29, p < 0.0001).

**Conclusions:**

Our study supports the concept of circulating EVs as a tool for non-invasive diagnosis of ovarian cancer. EVs also play pivotal roles in coagulation process, implying the inherent mechanism of generation of thrombus which often occurred in ovarian cancer patients at late stages.

## Background

Epithelial ovarian cancer (EOC) is the most lethal cancer among gynecological malignancies [1]. Almost 70% of EOC patients are diagnosed at an advanced stage. Although cytoreductive surgery followed by platinum/taxane-base chemotherapy has significantly improved the overall survival of EOC patients, the 5-year survival rate of EOC patients is approximately 45% [2]. Lack of an effective screening approach for early diagnosis is one of the main reasons for the high mortality. Serum CA125 and ultrasonography are mainstream applied methods accepted clinically in ovarian cancer diagnosis. However, due to the non-specificity of CA125, malignant diseases cannot be distinguished from benign diseases, such as inflammatory situations [3]. Extracellular vesicles (EVs) are small (40-1000 nm) membrane-enclosed microvesicles that play an important role in intercellular communication, involved in multifaceted physiological and pathological activities, including coagulation, angiogenesis, cell survival, modulation of the immune response, and inflammation [2, 4, 5]. Circulating EVs emanate from multiple cells, such as platelets, inflammatory cells, monocytes/macrophages and ovarian cancer cells. As circulating EVs carry complex biological information from their donor cells [6–9] and can be obtained using non-invasive approaches [2], they are emerging as a new type of cancer biomarker. Some studies have focused on ovarian cancer derived EVs for the potential of serving as biomarkers. Claudin-4 containing exosomes can be detected in the peripheral circulation of ovarian cancer patients, serving as a promising biomarker in ovarian cancer [10]. An exosomal microRNA signature consisting of eight miRNAs (miR-21, miR-141, miR-200a, miR-200c, miR-200b, miR-203, miR-205 and miR-214) was also investigated to be a potential diagnostic tool for ovarian cancer [11]. Besides, proteomic analysis of ovarian cancer cell derived exosomes also proved that exosomal protein present some tissue-specific protein signature which provide a potential source of blood-based protein biomarkers [12]. Despite the progress achieved in several studies regarding exosomal contents in cell lines as diagnostic markers, few studies focused on a systemic proteomic analysis and biological function of serum EVs derived from ovarian cancer patients. Systematic proteomics analysis of serum EVs derived from ovarian cancer patients can not only provide a more comprehensive understanding of EV proteins in clinic, but also lay the foundation of further studies exploring the mechanism of action of EVs in tumorigenesis, metastasis, relapse and so on.

With the development of technology of proteomic analysis, isobaric tags for relative and absolute quantification (iTRAQ) labeling coupled liquid chromatography-mass spectrometry (LC-MS) are newly emerging technologies that provide more information compared with conventional technologies [13]. In this study, we systemically investigated circulating EV proteins in ovarian cancer and healthy states using iTRAQ labeling coupled LC-MS, aiming to identify the differentially expressed proteins and to investigate their biological functions and also the potential of diagnostic biomarkers.

## Methods

### Subjects and serum sample collection

EOC serum samples (1.5 ml) were obtained from the tissue banks of Peking Union Medical College Hospital (PUMHC, Beijing) and Fudan University Shanghai Cancer Center (FUSCC Shanghai). All samples were obtained before surgery from patients without any prior treatment. All EOC patients (*n* = 70) were diagnosed at an advanced stage (stage III–IV) after primary cytoreductive surgery, and all of them were pathologically confirmed. Healthy controls (n = 70) were age-matched female volunteers with no cancer detected. For each group, 10 samples were used for proteomic analysis and 20 samples were used for Elisa validation of the proteomic results. Another cohort of 40-paired samples was prepared for the validation of the biomarker potential of candidate proteins. All serum samples were stored at − 80 °C. Informed consent was obtained from all participants, and this study was approved by the Ethical Committees of Peking Union Medical College Hospital and Fudan University Shanghai Cancer Center.

### Circulating extracellular vesicle isolation and identification

Circulating EVs were isolated using ExoQuick®, a commercial exosome precipitation reagent (Systems BioSciences, Inc. Mountain View CA), following the manufacturer’s protocol [14]. In brief, serum samples from individual patients and controls were centrifuged at 12,000×g for 10 min at 4 °C. The supernatant was then filtered through a 0.22-μm filter (MillPore, Billerica, MA, USA). Four volumes of supernatant was incubated with one volume of ExoQuick® buffer for 30 min at 4 °C. The mixture was centrifuged at 1500×g for 30 min at 4 °C. The flow-through was collected and resuspended the pellets in 200 μl of 1 × PBS and stored at − 20 °C.

Electron microscopy (EM), western blotting and nanoparticle tracking analysis (NTA) were applied for EVs characterization using a previously established method [12, 15]. For EM, about 50 μl of prepared EVs were loaded to Formvar carbon-coated 200-mesh copper grids and dried out. Then the absorbed exosome was negatively stained with 3% phosphotungstic acid and dried at room temperature. Next a transmission electron microscope (Olympus Software Imaging Solutions) was applied for observation at 120.0 kV and images were captured by a digital camera. Size and concentration of isolated extracellular vesicles were quantified by a NanoSight NS500 instrument (NanoSight, Amesbury, UK) using a previously established method [15]. Isolated serum extracellular vesicles were diluted into concentration from 2 × 10^8^ to 2 × 10^9^/ml. NanoSight software was stetted as follows: detection threshold, 9–10; blur, auto; and minimum expected particle size, 10 nm, and all of these settings were kept constant among all samples. Particle size and concentration were analyzed by the equipped NTA 2.0 software. For western blotting, two commonly used markers, ALIX and TSG101 (ProteinTech group, polyclonal, rabbit), were used [12]. Thirty microliters of isolated EV protein were loaded on 12% SDS-PAGE gels. Separated proteins were transferred to a polyvinylidene fluoride (PVDF) membrane, and then the PVDF membrane was blocked with 5% milk in 1× tris-buffered saline with Tween (TBST) (1 × 140 TBS with 0.05% Tween 20) for 1 h at room temperature. Next the membrane was incubated in primary antibodies at 4 °C overnight. Then the membrane was washed in TBST and incubated with horse radish peroxidase (HRP)-conjugated secondary antibody for 1 h at room temperature. An enhanced chemiluminescence (ECL) system (Thermo) was used to detect the blots.

### iTRAQ-LC-MS/MS analysis

#### Protein digestion and iTRAQ labeling

The prepared proteins were reduced with 10 mM DTT at 56 °C for 1 h and with 55 mM IAM in the dark at room temperature for 1 h. After adding 400-μl precooled acetone at − 20 °C for 3 h, the samples were centrifuged at 20,000×g for 30 min at 4 °C. After discarding the supernatant, the deposit was resuspended with 300 μl buffer (50% TEAB, 0.1% SDS). The prepared proteins were run on a short 10% SDS-PAGE gel and the gel was stained with Coomassie Blue G-250. EV protein lysate samples were 100 μg, and equal each sample volume with TEAB containing 1% SDS. Trypsin (3.3 μg) was added and incubated at 37 °C overnight. The digestion solution was lyophilized with 30 μl TEAB.

The peptides were labeled with an 8-plex iTRAQ Multiplex Kit according to the manufacturer’s instructions (AB Sciex, Foster City, CA, USA) [16]. The normal control and EOC groups were individually labeled. Then the labeled samples were mixed equally and dried by vacuum centrifugation.

#### Mass spectrometry

The mixed labeled samples were analyzed by nano LC-MS/MS with a HPLC-RP column (Phenomenex, Luna 5u C18(2), 100 mm × 75 mm). Peptides were loaded on a trapping column and over a 75-μm analytical column at 400 nL/min using a 65-min reverse phase gradient [14]. Resulting peptide and fragmentation spectra were input into software PD (Proteome Discoverer 1.3, Thermo), and analyzed using a Mascot database (Matrix Science, London, UK; version 2.3.0). In this study, 1.2-fold change (upregulation or downregulation) was used as a cut-off for biological significance based on the standard deviation and normalized peptide ratios [17].

#### Bioinformatics analysis

All differentially expressed proteins were searched in the PANTHER database (http://www.pantherdb.org/) [18]. Protein classification was based on functional annotations. The Ingenuity Pathway Analysis (IPA, Qiagen, USA) database was applied for pathway analysis. The accession numbers of identified proteins were submitted to the IPA with stetted *p*-values and fold changes. Canonical pathways, biological functions and networks of interconnected proteins were analyzed.

#### Validation of proteins using ELISA

Twenty samples were used for validation in each EOC group and control group. Candidate protein levels were determined using an ELISA kit from SAB Inc. according to the manufacturer’s instructions.

#### Factor X chromogenic activity assay

Coagulation was accessed by determining Factor X activity. The Factor X chromogenic activity assay (Abcam, MA, USA) measures the activation of zymogen Factor X to Factor Xa by RVV. Factor Xa as the activator of prothrombin occupies a central position linking the two blood coagulation pathways. The assay was conducted according to manufacturer’s manual. In brief, all reagents, samples and standards were prepared as instructed. EVs were extracted from 400 μL serum. 20 μL of Factor X standard or samples was added into the plate. 40 μL of freshly prepared Assay Mix was then added and mixed well by shaking. The UV absorbance at 405 nm was recorded every 2 min for 10 min by a plate reader (Thermo Fisher, MA, USA). The changes in absorbance per minute and standard concentrations were utilized to generate a standard curve. The unknown sample concentration was determined from the standard curve and multiplied by the dilution factor.

### Statistical analysis

All the quantitative measurements were triplicate. Student’s *t* test and Mann-Whitney U were used for comparison and a *p* value < 0.05 was considered as a significant difference. AUC curve were performed with SPSS and MedCalc using ROC analysis.

## Results

### Isolation and identification of circulating extracellular vesicles

**Fig. 1:**
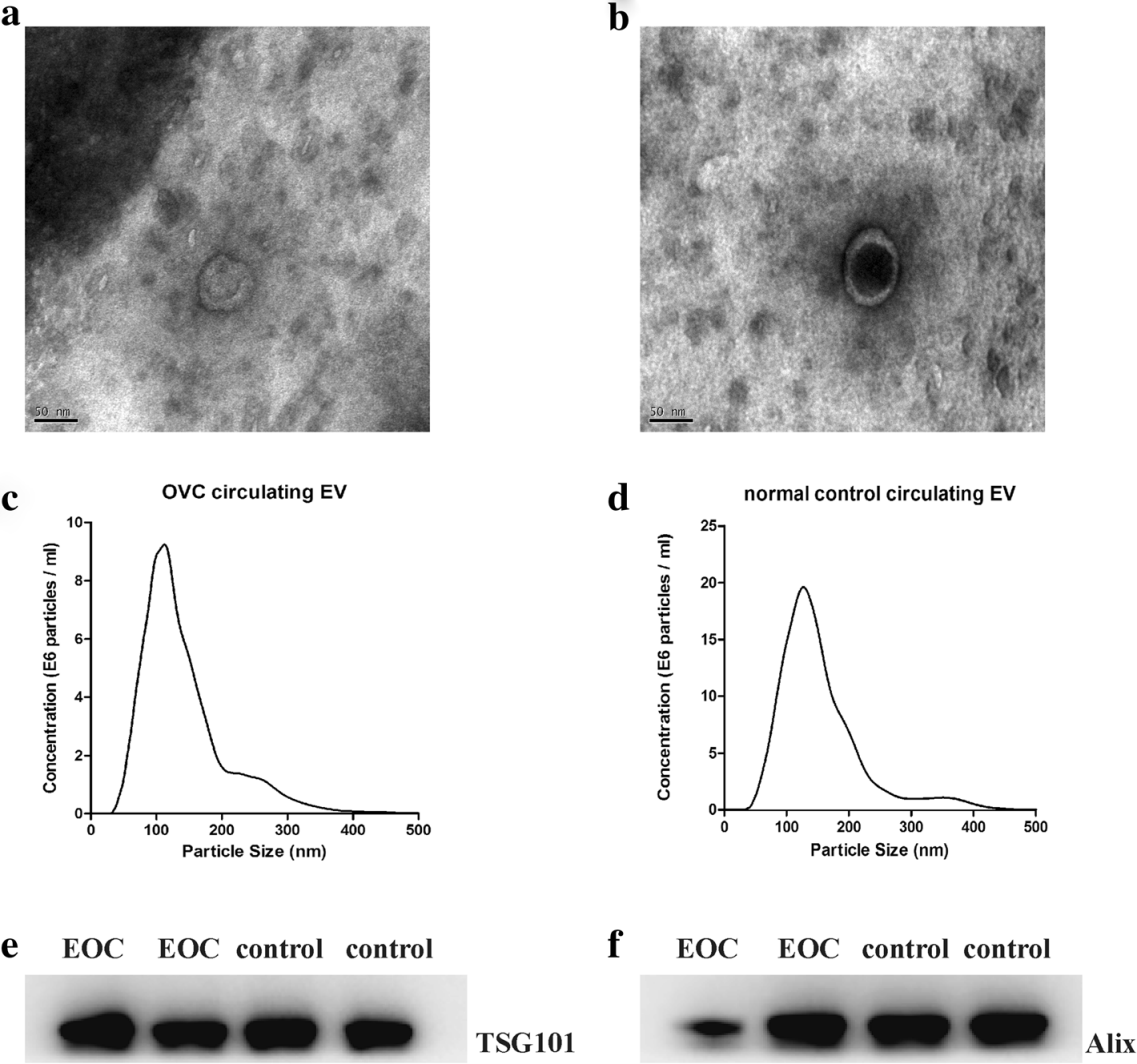
Identification of circulating EVs from EOC and control group by TEM, NTA and WB. **a** and **c** show EOC EVs identified by TEM and NTA. **b** and **d** shows circulating EVs identified by TEM and NTA from control group. **e** and **f** show EVs identified by WB using commonly used biomarkers TSG101 and Alix. Typical shape, size, size distribution and biomarkers of EVs were detected

Isolated circulating EVs were characterized by EM, western blotting and NTA (Fig. 1). Typical size, shape and protein markers were well defined, which indicated that circulating EVs from both EOC patients and controls were successfully isolated with high quality.

### Differentially expressed proteins and ingenuity pathway analyses

Clinical characteristics of the patients recruited for proteomics analysis were shown in Table 1. Details of clinicopathology data of all those patients were shown in Additional file 2: Table S1. Proteomic analysis of circulating EVs from the EOC group and controls totally yielded 1913 proteins (Additional file 2: Table S1) and 408 significantly differentially expressed proteins (Additional file 3: Table S2). Compared with normal controls, 200 proteins were upregulated and 208 proteins were downregulated in EOC group. Cellular component, biological process and molecular functions of differentially expressed proteins were analyzed (Additional file 1: Figure S1A, B, C). Results indicated that most components were from extracelluar region, and have receptor activity, which was in concordance with the origins of these proteins.

**Table 1:** Clinical characteristics of the patients recruited for proteomics analysis

Number	Age range (year)	CA125 (0.00–35.00 U/ml)	HE (40–81.9 pmol/L)	Prothrombin time (11–14.5 s)	CRP (0.0-5 mg/L)	Histopathology	FIGO stage
1	43–72	120.1	365.9	12.4	4.6	HGSC	IIIC
2	43–72	103.7	160.4	12.7	NA	HGSC	IIIC
3	43–72	39.08	94.96	13	NA	HGSC	IIIA
4	43–72	> 5000	1400	12.7	NA	HGSC	IIIC
5	43–72	239.6	> 1500	13.6	NA	HGSC	IIIC
6	43–72	188.3	499.3	12.3	NA	HGSC	IIIC
7	43–72	1104	590.3	12.8	NA	HGSC	IV
8	43–72	70.63	55.35	13	NA	HGSC	IIIC
9	43–72	422.3	102	12.7	NA	HGSC	IIIC
10	43–72	381	52.09	12.8	NA	HGSC	IIIC

**Fig. 2:**
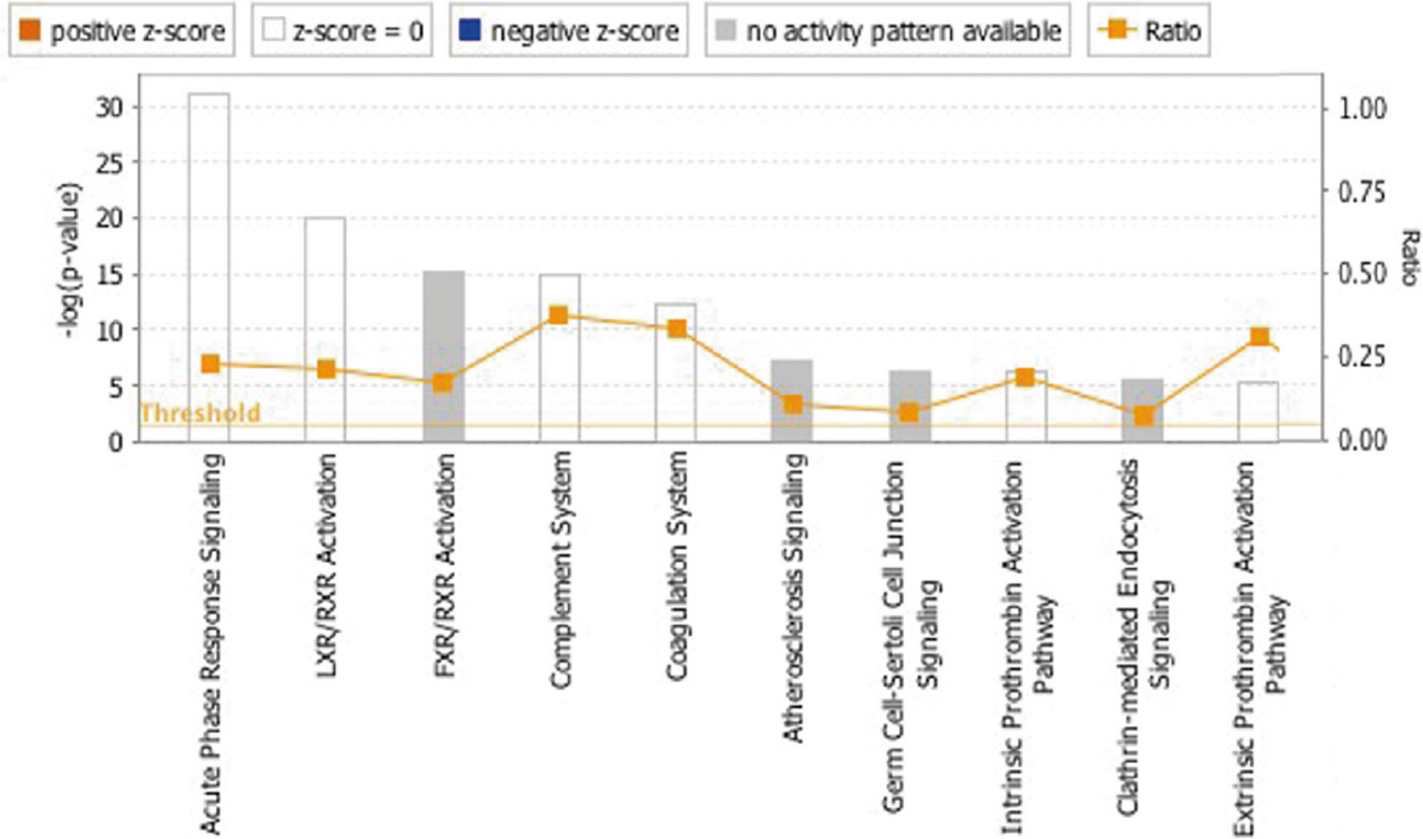
Canonical pathway analysis of the differentially expressed proteins. Five related pathways were identified, namely acute phase response signaling pathway, LXR/RXR activation, FXR/RXR activation, the complement system and the coagulation system

IPA analysis was used to further analyze the functions and interaction among these differently expressed proteins. The disease and biological function analysis revealed that most differentially expressed proteins were involved in inflammatory response, metabolic disease, cardiovascular disease, hematological disease and organism injury and abnormalities (Table 2). According to canonical pathway analysis, five related pathways and three networks were identified. The five pathways comprised the acute phase response signaling pathway, LXR/RXR activation, FXR/RXR activation, the complement system and the coagulation system (Fig. 2). It is generally accepted that gynecological cancers are associated with a high rate of thromboembolism, especially in ovarian cancer. Therefore, special attention was paid to the complement and coagulation pathway for further study.

**Table 2:** Disease and biological function analysis of differently expressed proteins

Top Diseases and Bio Functions		
Diseases and Disorders	p-value	Molecule
inflammatory Respons	3.76E-04 - 1.87E-19	143
Metabolic Disease	3.24E-04 - 9.58E-13	108
Cardiovascular Disease	3.24E-04 - 1.61E-13	86
Hematological Disease	3.24E-04 - 1.61E-13	67
Organismal Injury and Abnormalities	3.71E-04 - 1.61E-13	371

Three significant networks identified were: Network1, RNA Post-Transcriptional Modification, Cancer, Cell Death and Survival (p-score 51); Network2, Humoral Immune Response, Inflammatory Response, and Hematological Disease (p-score 34); Network3, Cellular Assembly and Organization, Cellular Function and Maintenance, Cell-To-Cell Signaling and Interaction (p-score 34).

Twenty-three focused molecules, including serpin C1 and C1q in Network 2 and another 23 proteins from Network3 were selected for further analysis.

### Biomarker potential of candidate biomarkers and promote coagulation activation

**Fig. 3:**
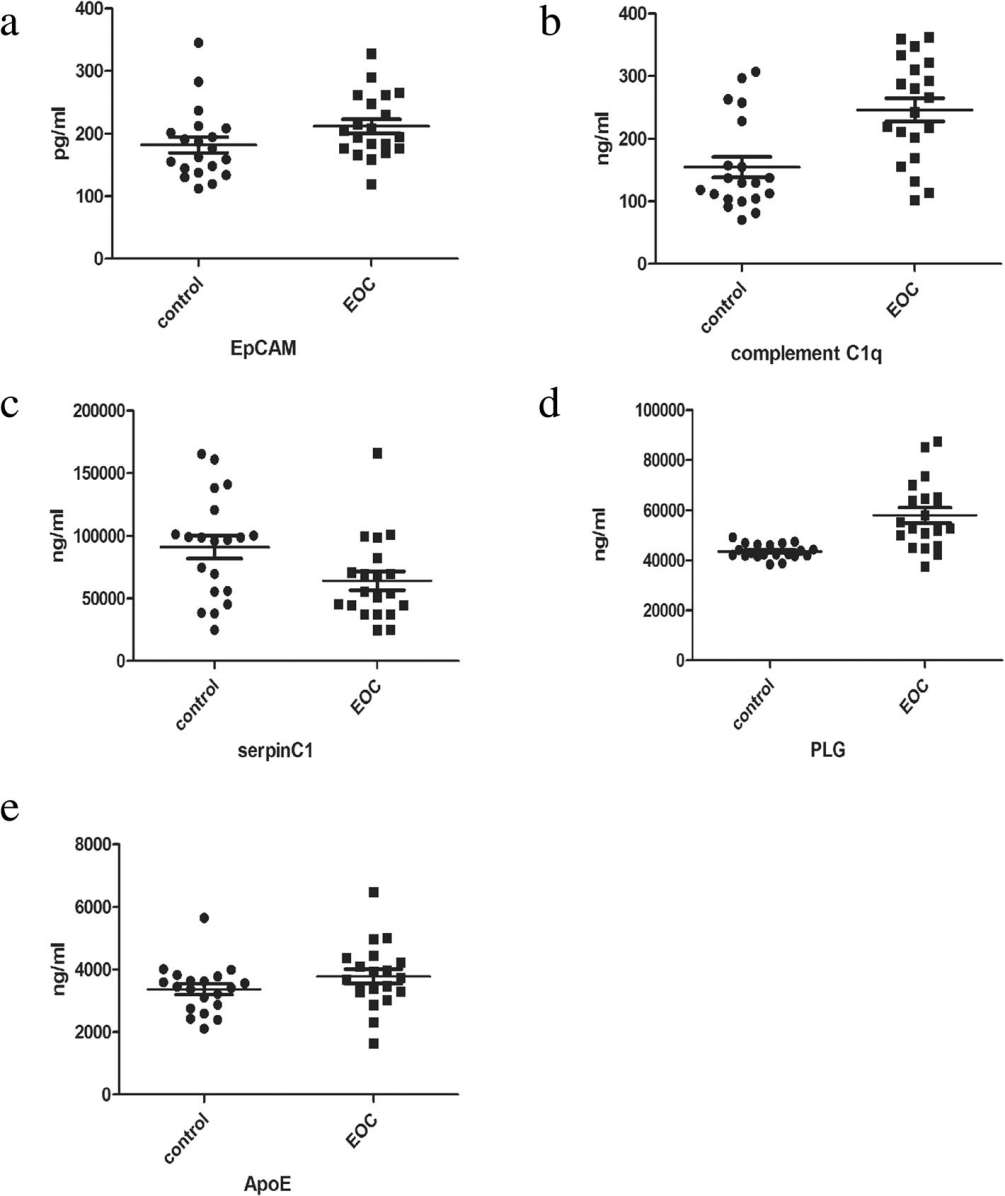
Elisa validation of expression levels of the five biomarkers. Expression level of EpCAM (**a**), C1q (**b**), serpinC1 (**c**), Plg (**d**) and ApoE (**e**) are significantly different in EOC and control group. All *p* value < 0.05

Clinical characteristics of patients with epithelial ovarian cancer recruited for ELISA was presented in Table 3. Four overexpressed proteins present in the EOC group, including EpCAM, C1q, ApoE and Plasminogen (plg) were chosen as the candidate markers for the validation of diagnosis evaluation. Serpin C1 was selected because it was significantly downregulated in the EOC group. Besides, one study suggested that ApoE is associated with intraluminal vesicles (ILV) within endosomes and remains associated with ILVs when they are secreted as EVs [19]. Moreover, plasminogen (plg) was proved to be a favorable biomarker for prediction of survival in advanced high-grade serous ovarian cancer [20]. Epithelial cellular adhesion molecule (EpCAM) was also proposed as a cancer-related factor in other malignancies. ELISA assay was applied for the quantification of protein levels in validation cohort. The expression profile of these four proteins in ELISA resembles that in proteomic analysis, showing a similar trend. The expression levels of EpCAM, plg, ApoE, serpinC1 and C1q in EOC group were 119.83 ng/ml, 58,127.48 ng/ml, 3716.77 ng/ml, 54,949.01 ng/ml and 254.41 ng/ml respectively; while the expression levels of EpCAM, plg, ApoE, serpinC1 and C1q in the control group were 112.65 ng/ml, 43,634.99 ng/ml, 3232.29 ng/ml, 97,900.40 ng/ml and 129.72 ng/ml. Expression levels of the five biomarkers in the EOC group and the control group were significantly different (all *p* < 0.05) (Fig. 3). These results were consistent with the results obtained by the proteomic analysis.

**Table 3:** Clinical characteristics of patients with epithelial ovarian cancer recruited for ELISA

Number	CA125 (0.00–35.00 U/ml)	HE (40–81.9 pmol/L)	Prothrombin time (11–14.5 s)	DDI(0.00–0.5μg/ml)	CRP (0.0-5 mg/L)	Histopathology	FIGO stage	Postoperative residual tumor
1	190.4	244.3	12.2	0.96	NA	EEC	IIIB	0
2	1586	233.9	14.8	> 20.00	NA	HGSC	IIIC	< 1 cm
3	> 5000.00	855.6	13.1	9.59	NA	HGSC	IIIC	0
4	> 5000.00	> 1500	13.2	5.38	32.1	HGSC	IIIC	NA
5	752.1	465.1	12.3	0.75	NA	HGSC	IIIC	< 0.5 cm
6	2195	> 1500	13.9	6.97	NA	HGSC	IIIC	> 1 cm
7	49.74	72.88	12.7	0.31	NA	HGSC	IIIB	0
8	1614	324.5	13.6	4.33	NA	HGSC	IV	< 1 cm
9	289.3	468.3	13.6	13.57	NA	HGSC	IIIC	< 0.5 cm
10	188	247.3	13.4	7.47	NA	HGSC	IIIC	< 0.5 cm
11	1451	378	12.4	4.62	22	HGSC	IVa	< 0.5 cm
12	> 5000	905.9	12.6	> 20.00	NA	HGSC	IVB	0
13	2753.00	214.70	12.7	3.55	NA	HGSC	IIIC	> 2 cm
14	1148	716.1	13.8	3.99	NA	HGSC	IIIB	0
15	572.4	678.6	12.8	4.20	NA	HGSC	IIIC	0.3 cm
16	646.7	305.3	13.1	10.7	35.7	HGSC	IIIb	0
17	671.4	63.85	13.5	3.45	NA	adenoca	NA	NA
18	> 5000	705.2	13.4	5.93	NA	HGSC	IIIA1	0
19	> 5000	> 1500	13.4	7.51	NA	HGSC	IVA	> 1 cm
20	511.8	> 1500	12.8	11.64	NA	HGSC	IIIC	0
21	> 5000	448.1	12.4	7.31	< 0.3	HGSC	IIIC	0
22	1296	562.2	12.7	6.74	15.4	HGSC	IIIC	0
23	235	180	13.5	2.8	16.1	CCC	IV	> 2 cm
24	1191	324.5	12.6	6.93	NA	HGSC	IIIC	> 1 cm
25	1137	> 1500	14.2	17.51	NA	HGSC	IIIC	< 0.2 cm
26	600.5	1168	12.5	16.27	NA	HGSC	IIIC	0
27	> 5000	633.2	14.9	4.53	NA	HGSC	IVB	0
28	2276	369.6	13.1	3.29	NA	HGSC	IVB	0
29	553.9	291	12.8	3.67	NA	HGSC	IV	0
30	> 5000	543.1	14	7.87	NA	HGSC	IIIC	0
31	1700	> 1500	13.9	14.99	NA	HGSC	IIIC	0
32	8.28	179.6	13.4	0.66	5.6	HGSC	IIIB	0
33	317.7	172.3	13.6	1.33	NA	HGSC	IIIC	0
34	2324	345.1	13.6	4.49	NA	HGSC	IIIC	> 2 cm
35	2661	> 1500	13.9	4.37	NA	HGSC	IVA	0
36	511.1	1221	13	12.26	NA	HGSC	IIIC	0
37	2430	229.5	14.2	6.08	19.9	HGSC	IIIC	0
38	1517	153.8	13.7	0.78	< 0.3	HGSC	IIIC	< 0.3 cm
39	921.2	788.6	13	7.36	NA	HGSC	IIIC	0
40	140.2	281.8	12.7	2.24	NA	HGSC	IIIC	0.1–0.2 cm
41	425.2	115.9	14.6	2.98	NA	HGSC	IIIC	< 1 cm
42	276	1436	14.3	2.64	38.4	HGSC	IIIC	0
43	1556	276.6	13.3	2.51	NA	HGSC	IIIB	0
44	1423	1015	13.2	5.73	6.5	HGSC	IIIC	0.1–0.2 cm
45	554.3	1265	14	6.15	18.8	HGSC	IIIC	0
46	885.1	208.2	12.7	11.29	NA	HGSC	IIIB	0
47	734.7	783.3	13	3.42	2.5	HGSC	IIIC	< 0.5 cm
48	793.6	179.1	14.8	9.24	74.1	HGSC	IIIC	0
49	280.5	329.6	12.2	17.61	NA	HGSC	IIIC	0
50	1343	> 1500	15.3	12.78	25.9	HGSC	IIIC	0
51	> 5000	131.9	13.4	7.87	NA	HGSC	IIIC	0
52	896.5	259	13	5.41	19	HGSC	IIIC	0
53	121	1236	13.3	3.49	NA	HGSC	IIIC	< 0.5 cm
54	728.2	326.5	13.2	1.95	2	HGSC	IIIB	0
55	816.9	> 1500	12.5	29.13	0.6	HGSC	IIIC	0
56	> 5000	291.5	12.6	1.36	38.3	HGSC	IIIC	0
57	2915	> 1500	12.2	6.21	NA	HGSC	IV	0.2
58	453.2	621	13.1	18.82	5.3	adenoca	IIIC	0
59	2253	251.8	12.7	2.05	1.2	HGSC	IIIC	< 1 cm
60	166.2	423.5	15.5	> 40	78.2	HGSC	NA	NA

*EEC* endometrial adenocarcinoma, *CCC* clear cell carcinoma, *HGSC* high gread serous carcinoma, *adenoca* adenocarcinoma

**Fig. 4:**
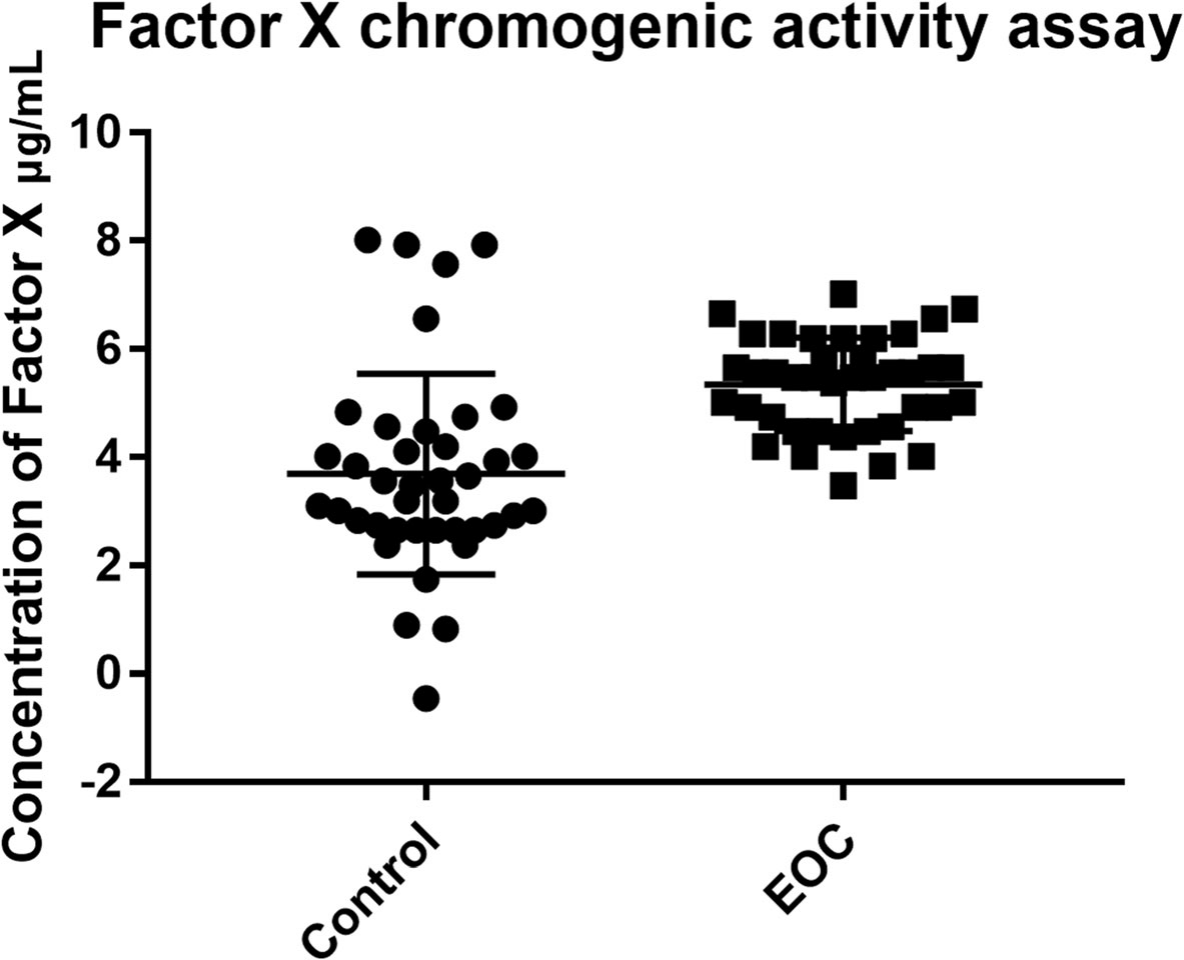
Activated Factor X in the EOC group and the control group. Activation of Factor X to Factor Xa was higher in EOC group than control (5.35 ± 0.14 vs. 3.69 ± 0.29, *p* < 0.0001)

**Fig. 5:**
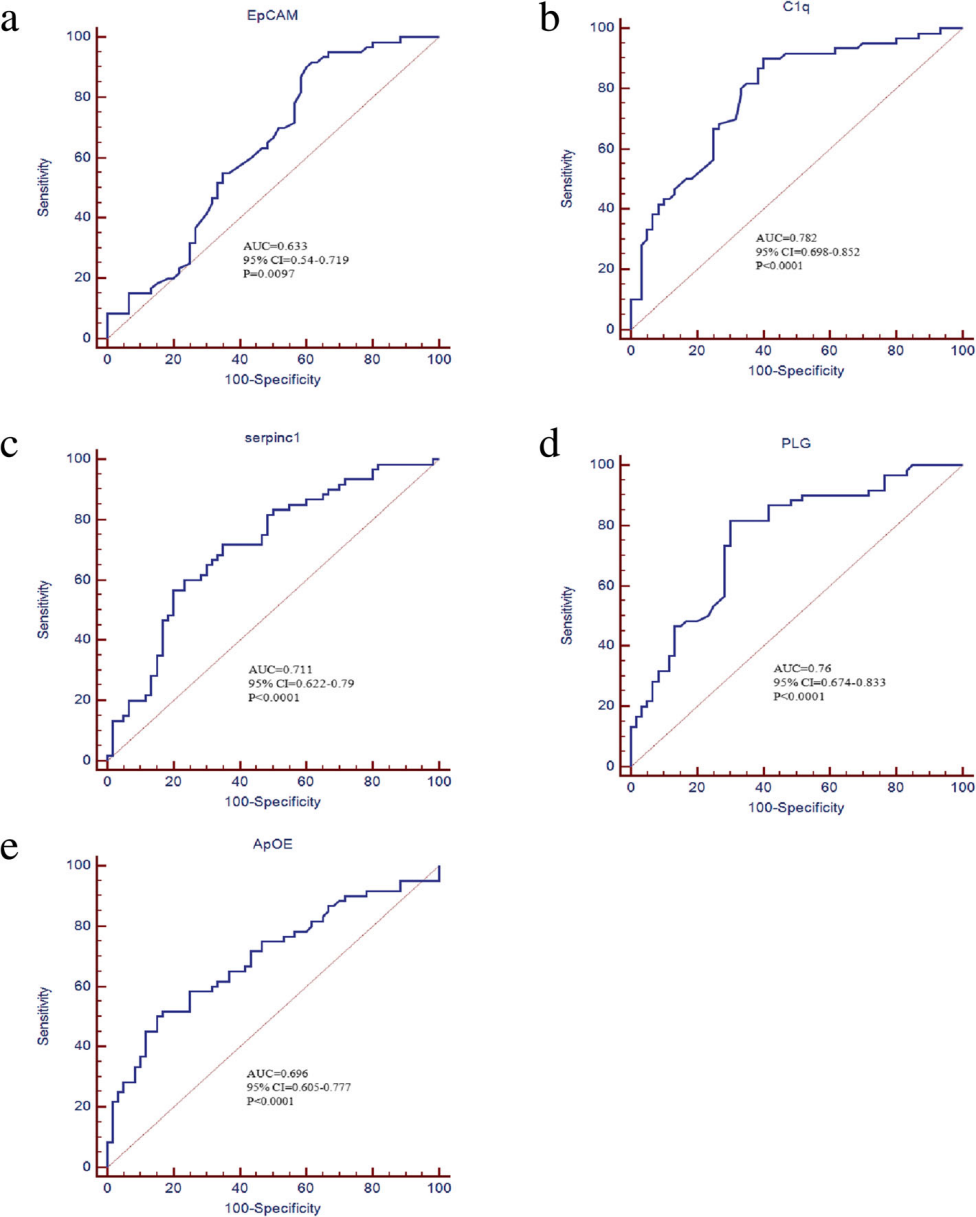
ROC curve analysis for EpCAM (**a**) Complement C1q (**b**), SerpinC1 (**c**), PLG (**d**), ApOE (**e**) all p value < 0.05, and AUC were list in each figure

**Fig. 6:**
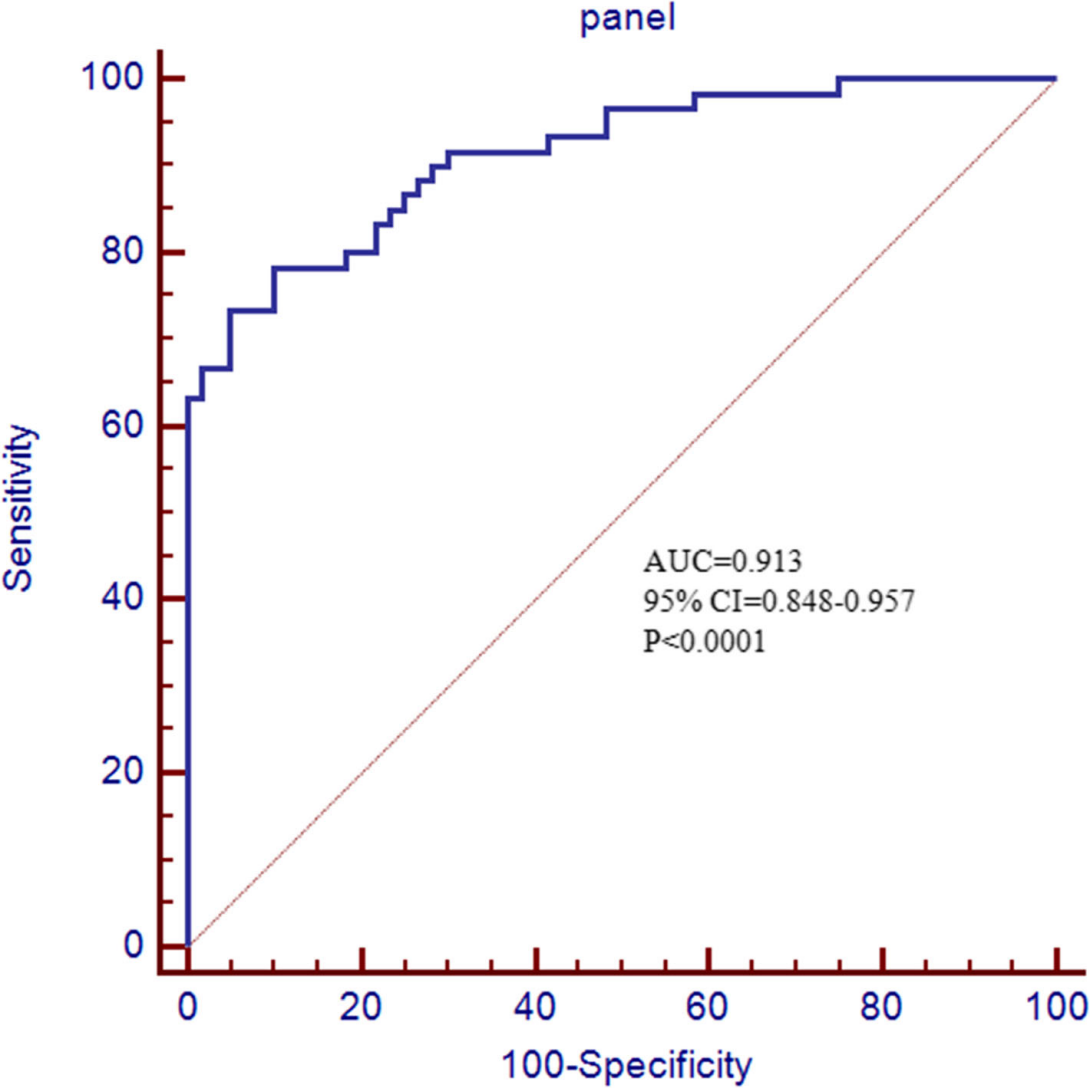
Multivariable logistic regression confirmed that ApoE multiplexed with EpCAM, plg, serpinC1 and C1q provide optimal diagnostic information for EOC with AUC = 0.913(95% confidence interval (CI) =0.848–0.957, p < 0.0001)

Activation of Factor X to Factor Xa was higher in EOC group than control (5.35 ± 0.14 vs. 3.69 ± 0.29, *p* < 0.0001) (Fig. 4). AUC curve of single biomarker of ApoE multiplexed with EpCAM, plg, serpinC1 and C1q were presented in Fig. 5. Multivariable logistic regression confirmed that ApoE multiplexed with EpCAM, plg, serpinC1 and C1q provide optimal diagnostic information for EOC with AUC = 0.913, (95% confidence interval (CI) =0.848–0.957, p < 0.0001) (Fig. 6).

## Discussion

Lack of highly specific and sensitive serum biomarkers is a major problem in early detection of ovarian cancer. The most commonly used biomarkers in ovarian cancer is CA125 with low specificity [3]. Tumor-derived EVs are emerging as a new type of cancer biomarker [2]. Some studies focused on exosomal proteins [10] or exosomal microRNAs [11] as diagnostic biomarkers. Compared with conventional specimens, EV biomarkers provide a non-invasive approach and higher specificity and sensitivity known as “liquid biopsy” [21]. In this study, we systemically studied serum EVs proteins and their biological functions in both ovarian cancer patients and healthy women. A commercially-available exosome precipitation reagent was applied for isolation because of its high efficiency. EM, NTA and western blotting were used for identification of isolated EVs. Typical shape, size and biomarkers were confirmed by those methods indicating that high quality and purity serum EVs were successfully obtained, which is the foundation for our subsequent systemic proteomic analysis. Using iTRAQ labeling coupled LC-MS provide more precise quantification, and finally 408 significantly differentially expressed proteins were identified and their biological functions were investigated. Canonical pathway analysis identified five related pathway and we paid special attention to the complement system and the coagulation system. Proteins involved in the two systems namely plg, C1q and serpinC1 were selected for validation. Besides, ovarian cancer tissue specific protein EpCAM and ApoE were also verified. Validation results were consistent with the results obtained by the proteomic analysis, and these also proved the reliability of our proteomic results. Furthermore, we confirmed serum EVs promote coagulation by using a Factor X chromogenic activity assay. ApoE multiplexed with EpCAM, plg, serpinC1 and C1q provide optimal diagnostic information for EOC.

ApoE is an ovarian cancer tissue specific protein which has been recently identified as a potential biomarker in ovarian cancer [22, 23]. It is frequently detected in ovarian serous carcinomas, and is also a prognostic marker in ovarian cancer patients [22]. It has been demonstrated that ApoE expression is elevated both in ovarian cancer cells [12] and in ovarian cancer tumor fluids [24]. Beside, ApoE is required for cell proliferation and survival in ovarian cancer [22]. Ovarian cancer cell derived exosomes also overexpress ApoE [12]. EpCAM is also considered as an ovarian cancer tissue specific protein which is used for isolation of ovarian cancer derived exosomes [11, 25]. By using a 3D novel engineered ExoProfile chip, diagnostic power of seven markers (EGFR, HER2, CA125, FRα, CD24, EpCAM, and CD9 plus CD63) were evaluated with AUC = 1 in ovarian cancer derived exosomes [26]. Although only 15 patients were enrolled in this study, results showed a promising prospect of diagnostic potential of circulating exosomes. Serum PLG was once detected in a rat model using iTRAQ technique, and potential as biomarker was investigated [27]. It was also demonstrated as a favorable biomarker for prediction of survival in advanced high-grade serous ovarian cancer [20]. In our study, both of exosomal ApoE, EpCAM and Plg were detected and verified, and this proved that ovarian cancer tissue associated proteins are expressed on serum EVs and this is the foundation for further investigation of their biomarker potential.

It is generally accepted that gynecological cancers are associated with a high rate of thromboembolism, especially in ovarian cancer [28, 29]. Therefore, we paid special attention to the complement and coagulation pathway. It has long been known that EVs play a role as regulators of the coagulation system in cancer [30]. Tissue factor (TF), which is expressed on non-vascular cells, is the main activator of the coagulation cascade. TF is largely expressed on monocyte/macrophage-derived microvesicles, including exosomes [31]. SerpinC1 is a inhibitor of TF [32], and as the expression level of serpinC1 was downregulated in the EOC group, the TF-dependent coagulation pathway was promoted. As coagulation factor X is a central component of the coagulation cascade, factor Xa as the activator of prothrombin occupies a central position linking the two blood coagulation pathways, we accessed coagulation by determining Factor X activity. Level of activated Factor X was significantly higher in EOC group than control, and this demonstrated that EOC derived circulating EVs promote coagulation. Complement C1Q is the defining component of the classical pathway as it forms the C1Q/C1RC1S complex [33]. A previous study demonstrated that malignant cell-derived EVs activated the complement system [34]. Our results showed differentially expressed circulating EV proteins were involved in complement system activation, and all EV proteins that were involved in the complement cascade were elevated in the EOC group. Compared with the control group, the expression levels of C1Q in EOC EVs were significantly elevated. All of these demonstrated that EOC circulating EVs from multiple cells play an important role in complement system activation. And those results might give information for the management and treatment of ovarian cancer patients.

Receiver operating characteristic (ROC) curve analysis indicated that the area under the curve (AUC) for EpCAM, plg, serpinC1 and C1q was statically significant. Multivariable logistic regression confirmed that ApoE multiplexed with EpCAM, plg, serpinC1 and C1q provide optimal diagnostic information for differentiating the EOC and control group with AUC = 0.913 (95% confidence interval (CI) =0.848–0.957, *p* < 0.0001). These results demonstrated that the panel of EV biomarkers might be more promising in ovarian cancer diagnosis than the individual biomarker.

In early ovarian cancer detection, biomarkers based on high-throughput technologies of proteomics have shown promise prospect in the past decades [3]. Specimen ranged from various body fluids, including utero-tubal lavage [35], tumor fluids [24], plasma [36] and even cell or circulating EVs [12, 37]. Compare with biomarkers detected in traditional specimens, exosomal biomarkers is more specific and sensitive due to their excellent stability [25]. Marcisauskas et al. [38] verified one biomarker panel with nine proteins in cystfluid and serum, and the biomarker panel achieved ROC AUC 0.96 and 0.57 respectively. Enroth et al. [36] identified a high-accuracy 11 plasma protein biomarker signature for ovarian cancer with an AUC 0.94. In our study, biomarker potential of a panel of five EV proteins was verified with an AUC 0.913. Compared with those studies, our serum EV protein biomarker panel performed better as we only enrolled 5 proteins and more noninvasive compared with cystfluid. Biomarker potential of exosomal Claudin-4 and microRNAs were also investigated, and our study provides a more comprehensive understanding of EV proteins in vivo which can provide more precise information for further study.

What type of “liquid fraction” of blood should be performed for analytical study is a constant debate. As serum is free of clotting proteins, cells and platelets, it is considered as the gold standard in many applications [39]. In our published data [37], biomarker potential and biological functions of plasma EV proteins were also investigated. Compared with serum EV proteins, 57 differentially expressed proteins were also detected in plasma EV proteins and most of them were involved in blood coagulation pathway and plasminogen activating pathway. By using different proteomic approaches and different blood fraction, we found that differentially expressed proteins are overlap in plasma and serum EVs, and most of them were involved in coagulation system. This demonstrated that circulating EVs play an important and universal role in coagulation in ovarian cancer. In this study, we established a protein database for serum EVs of ovarian cancer which is many differences as well as similarities compared with plasma EVs.

There was some limitations of our study, such as the small sample size for validation. What’s more, in this study, all the enrolled patients were diagnosed at advanced stage, and CA125 levels of all patients were elevated, we didn’t compare the performance of CA125 with this panel of biomarkers. Because more than 70% patients are diagnosed at advanced stage, we are enrolling more patients at early stage especially those with normal CA125 level to compare the performance of CA125 with our panel of biomarkers. Besides, we are enrolling more patients diagnosed as other benign conditions to validate the specificity of these biomarkers.

## Conclusion

We identified circulating EVs as a potential tool for non-invasive diagnosis of ovarian cancer which confirms the conception of circulating EVs as liquid biopsy biomarker in cancer. EVs also play pivotal roles in coagulation process, implying the inherent mechanism of generation of thrombus which often occurred in ovarian cancer patients at late stages. Our study shed light on the comprehensive profiling of EVs proteins in ovarian cancer, providing diverse aspects for further evaluation.

## Supplementary information

**Additional file 1: Figure S1A-C.** Cellular component (A), biological process (B) and molecular functions(C) of differentially expressed proteins.

**Additional file 2: Table S1.** Total protein identified by proteomic profiling.

**Additional file 3: Table S2.** Differential protein identified by proteomic profiling.

## Data Availability

The datasets used and analyzed during the current study are available from the corresponding author on reasonable request.

## Abbreviations

CI: Confidence interval
EM: Electron microscopy
EOC: Epithelial ovarian cancer
EVs: Extracelluar vesicles
FUSCC: Fudan University Shanghai Cancer Center
HRP: Horse radish peroxidase ECL Enhanced chemiluminescence
IPA: Ingenuity pathway analysis
iTRAQ: Quantitative proteomic analysis
LC-MS: Liquid chromatography-mass spectrometry
NTA: Nanoparticle tracking analysis
PD: Proteome discoverer
plg: Plasminogen
PUMHC: Peking Union Medical College Hospital
PVDF: Polyvinylidene fluoride
TBST: Tris-buffered saline with Tween
ILV: Intraluminal vesicles
EpCAM: Epithelial cellular adhesion molecule
AUC: Under the curve
ROC: Receiver operating characteristic
TF: Tissue factor
PSGL-1: P-selectin glycoprotein ligand-1
PMV: Platelet-derived microvesicles
TLRs: Toll-like receptors
