# Supplementary figures and images for: Ovarian cancer circulating extracelluar vesicles promote coagulation and have a potential in diagnosis: an iTRAQ based proteomic analysis
Source: BMC Cancer. 2019 Nov 12;19:1095. doi: 10.1186/s12885-019-6176-1 (PMC6852975; doi:10.1186/s12885-019-6176-1)

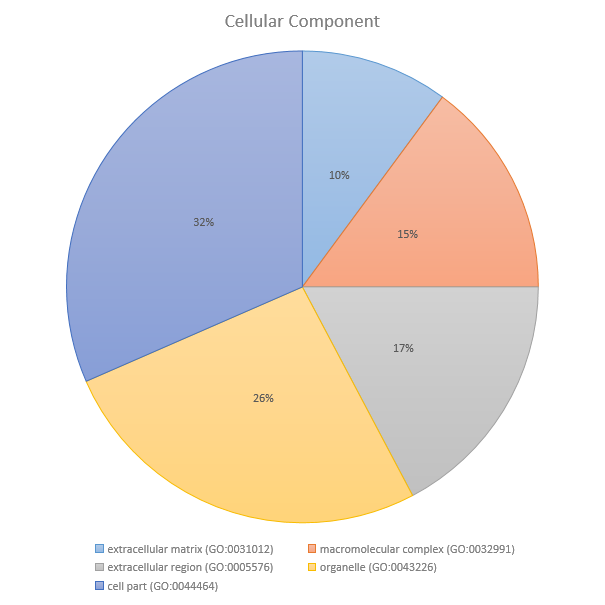

Supplement: Supplementary file 1 — Additional file 1: Figure S1A-C. Cellular component (A), biological process (B) and molecular functions(C) of differentially expressed proteins. [file 12885_2019_6176_MOESM1_ESM.zip › Fig.S1A.tif]

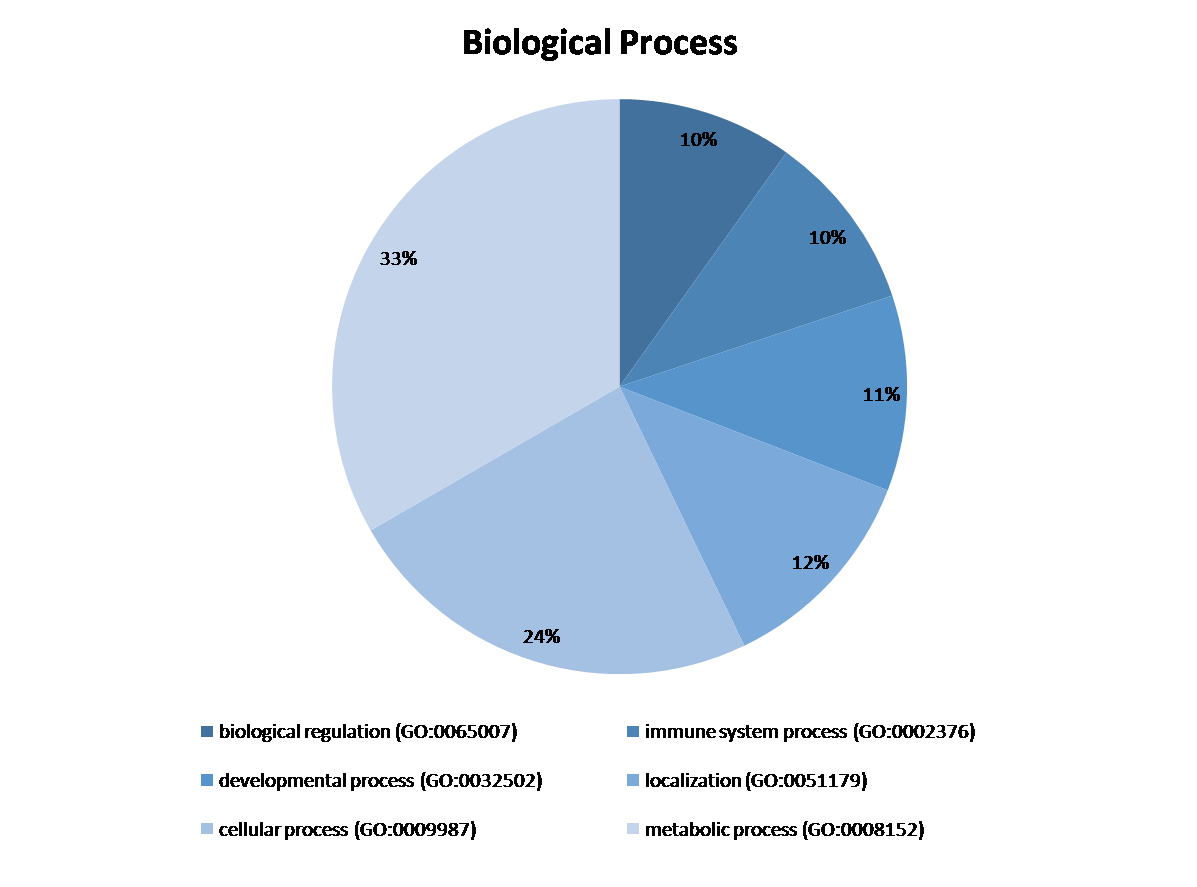

Supplement: Supplementary file 1 — Additional file 1: Figure S1A-C. Cellular component (A), biological process (B) and molecular functions(C) of differentially expressed proteins. [file 12885_2019_6176_MOESM1_ESM.zip › Fig.S1B.tif]

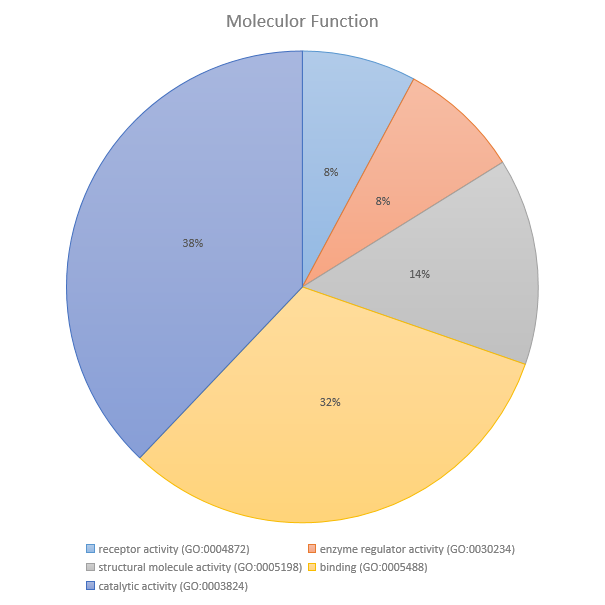

Supplement: Supplementary file 1 — Additional file 1: Figure S1A-C. Cellular component (A), biological process (B) and molecular functions(C) of differentially expressed proteins. [file 12885_2019_6176_MOESM1_ESM.zip › Fig.S1C.tif]
